# Role of gut microbiota and immune response in breast cancer progression

**DOI:** 10.17305/bb.2025.12003

**Published:** 2025-04-14

**Authors:** Xiaofang Zhang, Na Ma, Conghui Jin, Xiaoli Cao

**Affiliations:** 1 Department of Infection Management, Affiliated Cancer Hospital of Nantong University, Nantong Cancer Hospital Nantong, Jiangsu Province, China; 2 Department of Clinical Medicine, Medical School, Nantong University, Nantong, Jiangsu Province, China; 3 Department of Medical Oncology, Nantong University Affiliated Tumor Hospital, Nantong, Jiangsu Province, China; 4 Department of Clinical Laboratory, Nantong Cancer Hospital, Affiliated Cancer Hospital of Nantong University, Nantong, Jiangsu Province, China

**Keywords:** Gut microbiota, immune cells, breast cancer, Mendelian randomization, MR, causal inference

## Abstract

Breast cancer is one of the most prevalent cancers among women and is associated with high mortality rates. Emerging evidence suggests a link between gut microbiota and the development of various tumors, particularly those involving immune-mediated mechanisms. However, the potential relationship between gut microbiota and breast cancer—and whether this relationship is mediated by immune cells—remains unclear. This Mendelian randomization (MR) study utilized summary statistics from genome-wide association studies of 412 gut microbiota, 731 immune cell traits, and breast cancer (including its subtypes). Two-sample MR analyses were conducted to assess potential causal relationships between gut microbiota and breast cancer. To further validate the findings, Bayesian weighted MR was applied. Robustness was ensured through sensitivity, specificity, and pleiotropy analyses. A reverse MR analysis was also performed to assess the potential for reverse causality. Finally, mediation analysis was employed to investigate whether immune cells mediate the pathway from gut microbiota to breast cancer. The MR analysis identified 15 gut microbiota and related metabolic pathways significantly associated with breast cancer, with nine showing positive associations and six showing negative associations. The reverse MR analysis did not support a causal effect of breast cancer on gut microbiota. Mediation analysis revealed that DP (CD4^+^CD8^+^) % leukocyte mediated the pathway between gut microbiota (PWY-6263: superpathway of menaquinol-8 biosynthesis II) and breast cancer. These findings suggest a causal relationship between gut microbiota and breast cancer, with a small portion of this effect mediated by immune cells. This study underscores the potential role of gut microbiota and immune modulation in the pathogenesis of breast cancer.

## Introduction

Breast cancer is one of the most commonly diagnosed cancers in women and ranks as the second leading cause of cancer-related death among women [1, 2]. Approximately one in eight women in the United States will be diagnosed with breast cancer during their lifetime [3]. The risk of developing breast cancer increases with age, rising by about 0.5% per year [4]. Previous studies [5] have suggested that the high incidence of breast cancer in some developed countries may be associated with both lifestyle and genetic factors, such as BRCA1 and BRCA2 mutations [6], as well as diet and obesity [7]. Treatment for breast cancer typically involves a combination of surgery, chemotherapy, radiotherapy, targeted therapy, and endocrine therapy. Although significant progress has been made in early detection and treatment, options for advanced breast cancer remain limited and are often associated with a high incidence of adverse side effects [8]. Despite ongoing advancements, the exact mechanisms underlying breast cancer development are still not fully understood. Therefore, further research is needed to better understand the causes of breast cancer and develop effective prevention strategies. The gut microbiota, which establishes itself in the human gastrointestinal tract from birth, evolves in tandem with the host’s growth and development. It exists in a symbiotic relationship with the host and plays a critical role in maintaining overall health. The gut microbiota and the host form a complex ecosystem wherein a healthy gut environment promotes the growth of beneficial microbes and prevents the colonization of harmful bacteria. Additionally, the gut microbiota regulates both local and systemic immune functions. Through long-term co-evolution, the microbial flora and its host have developed a close symbiotic relationship based on mutual adaptation and selection. The influence of the gut microbiota on immune function—both locally and systemically—has garnered increasing attention. Musso [9], Maynard et al. [10], Frosali et al. [11], and Fujisaka et al. [12] have suggested that the gut microbiota is involved in the regulation of metabolic and immune activities, playing a key role in maintaining microbial balance. Many diseases associated with altered gut microbiota are linked to impaired immune responses [13, 14]. Increasing evidence suggests a potential association between gut microbiota and cancer risk. If this relationship is indeed causal, targeting the gut microbiota may represent a promising strategy for cancer screening and prevention [15]. Mendelian randomization (MR) is a data analysis technique used in epidemiological studies to investigate causal relationships. It employs genetic variants that are strongly associated with exposure factors as instrumental variables (IVs) to assess their causal effects on specific outcomes. By minimizing the influence of residual confounding, MR provides stronger evidence for causality than traditional observational studies or randomized controlled trials [16]. Given the interaction between gut microbiota and tumors, we hypothesized that specific microbial taxa may increase the risk of breast cancer. To explore this, we conducted a two-sample MR study to investigate the potential causal relationship between breast cancer and 412 gut microbiota, and further identified relevant immune cells through mediation analysis. This study aimed to elucidate the mechanisms by which the gut microbiota contributes to the development of breast cancer, offering a new scientific foundation for personalized treatment strategies. In conclusion, this study explored the roles of gut microbiota, immune cells, and their interplay in breast cancer pathogenesis. By leveraging advanced genetic techniques and conducting a comprehensive analysis of immune cell signatures, the study identified potential therapeutic targets and contributed to a deeper understanding of the complex etiology of breast cancer. These findings may help reduce morbidity, recurrence, and mortality by guiding interventions targeting the gut microbiota and immune system to prevent breast cancer.

## Materials and methods

### Study design

**Figure 1.**
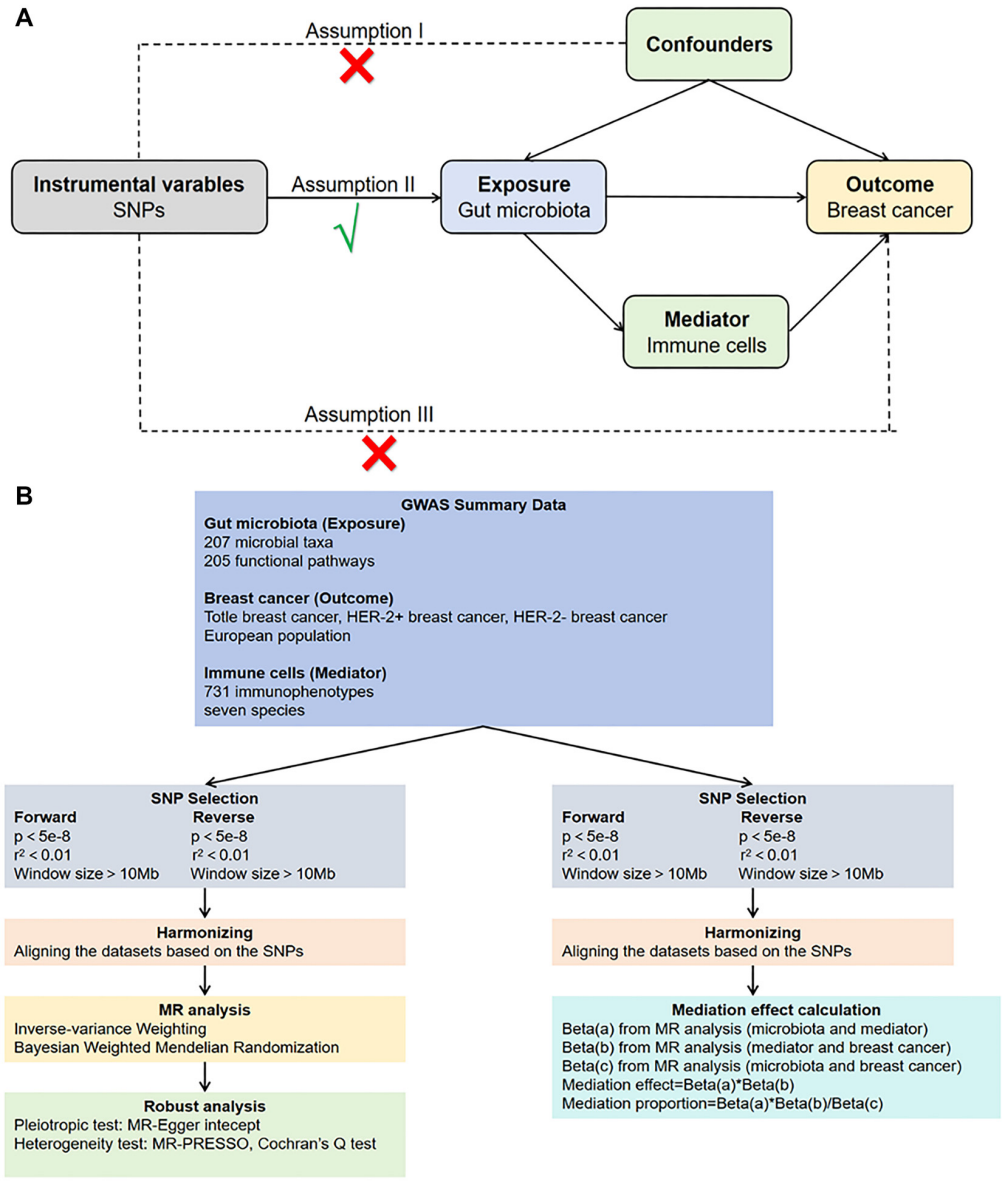
**Study design and flow chart.** (A) MR analysis principle; (B) Flow chart of the study. Abbreviations: MR: Mendelian randomization; SNP: Single-nucleotide polymorphism.

A bidirectional two-sample MR study design was used to investigate the causal relationship between 412 gut microbiota taxa (exposures) and the risk of breast neoplasia (outcome). The study followed four main steps. First, a two-sample MR analysis was conducted to assess the causal effects of gut microbiota on breast cancer and its subtypes, identifying taxa with strong associations. Second, the selected gut microbiota were further analyzed for their causal effects on specific immune cell types. Third, the relationship between the identified immune cells and breast cancer was evaluated. Finally, mediation analysis was performed to explore whether immune cells mediate the causal pathway from gut microbiota to breast cancer. The overall study design is illustrated in Figure 1. Genetic variants were used as IVs, with single-nucleotide polymorphisms (SNPs) from the FinnGen dataset serving this purpose. To ensure the validity of the IVs, three core assumptions were applied: (i) independence—SNPs are not associated with confounding factors; (ii) relevance—SNPs are strongly associated with the exposure; and (iii) exclusivity—SNPs influence the outcome only through the exposure. All data were derived from individuals of European ancestry and sourced from publicly available genome-wide association study (GWAS) databases. As the original studies obtained informed consent from participants, no additional ethical approval was required for this analysis.

### GWAS breast cancer

The GWAS data sources for breast cancer were obtained from the FinnGen R11 GWAS database, which integrates digital health records from the Finnish Health Registry with genetic data from the Finnish Biobank (https://www.finngen.fi/en). We used GWAS summary statistics from FinnGen for overall breast cancer (20,586 cases and 201,494 controls) as the primary outcome. Additionally, to investigate the association between gut microbiota and specific pathological subtypes of breast cancer, we obtained GWAS data for HER2-negative (8469 cases and 201,226 controls) and HER2-positive (12,081 cases and 201,226 controls) breast cancer from the same source. The UK Biobank was excluded from outcome data selection to avoid sample overlap between exposure and outcome datasets.

### Gut microbiota data sources for GWAS

SNPs associated with gut microbiota composition in this study were obtained from the NHGRI-EBI GWAS Catalog (https://www.ebi.ac.uk/gwas/) under study accession numbers GCST90027446–GCST90027857. Accession numbers for individual taxa and pathways are listed in Table S13 and available online at https://dutchmicrobiomeproject.molgeniscloud.org [17]. The GWAS data originate from the Dutch Microbiome Project, which includes 7738 participants and covers 207 microbial taxa and 205 functional pathways, representing microbial composition and function.

### Immune cells data sources for GWAS

The publicly available accession numbers, ranging from GCST90001391 to GCST90002121, contained an extensive array of 731 immunophenotypes [18]. These included data on the maturation phase of B cells, CDCs, T cells, monocytes, T-cell/B-cell/NK-cell assay, myeloid cells, and Treg cells [19]. The GWAS data contained four different types of 32 morphological parameters (MPs), 118 absolute cell counts (ACs), 192 relative cell counts, and 389 median fluorescence intensities, collected from 3757 European individuals with no overlapping cohorts [18].

### Selection of IVs

Significant SNPs of gut microbiota and immune cells with *P* < 1 × 10^-5^ and with linkage disequilibrium were excluded (*r*^2^ ═ 0.001, kb ═ 10,000) [20]. We clustered all genetic variants using a threshold of *R_2_* < 0.001 within a clustering distance of 10,000 kb. Subsequently, SNPs were filtered using the F-statistic method. The F-statistic was calculated for each IV, and SNPs with *F* > 10 were reserved for subsequent studies [21]. If the corresponding *F*-statistic is <10, IVs are considered as weak IVs and then excluded.

### Two-sample MR analysis

This study employed a two-sample MR analysis to investigate the causal relationship between gut microbiota and breast cancer. Causal effects were primarily assessed using the Inverse Variance Weighted (IVW) method, which calculates a weighted average of the causal effects of genetic variants based on the inverse of their variances. Additional methods—including MR-Egger, weighted median, simple mode, and weighted mode—were used as complementary approaches to validate the robustness of the findings.

### Statistical analysis

Sensitivity analyses were conducted to assess the reliability and stability of the conclusions, including heterogeneity analysis, horizontal pleiotropy analysis, and a “leave-one-out” test. Cochran’s Q statistic was used to evaluate heterogeneity. Horizontal pleiotropy was assessed using the MR-Egger intercept and the MR-PRESSO test. To evaluate the potential bias introduced by individual SNPs in the MR analysis, we performed a “leave-one-out” analysis by sequentially removing one SNP at a time and re-estimating the effect.

### Reverse MR analysis

To investigate whether breast cancer has a causal effect on the identified significant gut microbiota, we conducted a reverse MR analysis. In this analysis, SNPs associated with breast cancer were used as IVs, and the identified gut microbiota served as the outcome. The reverse MR approach was employed to help rule out potential bidirectional interactions between the exposure and outcome.

**Figure 2.**
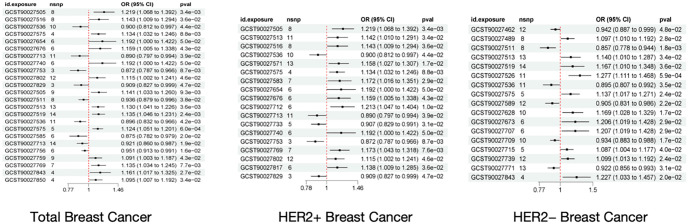
**The forest plot of the positive bacterial flora of breast cancer and its subtypes**.

**Table 1:** MR result of the association of gut microbiota with total breast cancer

**Exposure**	**SNPs**	**pval**	**or**	**or_lci95**	**or_uci95**	**Direction**
DAPLYSINESYN-PWY: L-lysine biosynthesis I	9	0.043	1.117	1.004	1.243	Positive
FAO-PWY: fatty acid beta oxidation I	7	0.021	0.895	0.815	0.984	Negative
METHGLYUT-PWY: superpathway of methylglyoxal degradation	8	0.044	1.059	1.002	1.120	Positive
PRPP-PWY: superpathway of histidine purine and pyrimidine biosynthesis	13	0.0179	1.118	1.019	1.226	Positive
PWY0-1298: superpathway of pyrimidine deoxyribonucleoside degradation	14	0.003	1.151	1.050	1.262	Positive
PWY-4984: urea cycle	8	0.031	0.862	0.753	0.986	Negative
PWY-5005: biotin biosynthesis II	11	0.001	0.883	0.819	0.951	Negative
PWY-6263: superpathway of menaquinol-8 biosynthesis II	5	0.007	1.110	1.029	1.196	Positive
PWY-6590: superpathway of *Clostridium acetobutylicum* acidogenic fermentation	6	0.025	0.866	0.763	0.982	Negative
PWY-7446: sulfoglycolysis	10	0.034	1.048	1.003	1.095	Positive
Pseudoflavonifractor	8	0.034	1.048	1.003	1.095	Positive
Lachnospiraceae noname	5	0.034	1.087	1.006	1.174	Positive
Roseburia	14	0.005	0.887	0.816	0.964	Negative
Parabacteroides merdae	4	0.011	1.204	1.043	1.390	Positive
Bacteroides intestinalis	3	0.040	0.915	0.841	0.996	Negative

Positive: Risk-increasing (OR>1); Negative: Risk-decreasing (OR<1). Abbreviations: pval: *P* value; OR: Odds ratio; or_lci95: Odds ratio lower confidence interval at 95%; or_uci95: Odds ratio upper confidence interval at 95%; MR: Mendelian randomization; FAO: Fatty acid β-oxidation.

### Mediation analysis

Mediation analysis was used to explore the potential mechanisms underlying the pathways from exposure to outcomes via mediation. First, β1 was obtained through two-sample MR analysis to evaluate the causal relationship between gut microbiota and immune cells. Next, two-sample MR methods were used to assess the causal relationship between the identified immune cells and breast cancer, yielding β2. The mediation effect was calculated by multiplying β1 by β2. To investigate the potential mediating role of immune cells in the pathway linking gut microbiota to breast cancer, we conducted multiple MR analyses. All analyses were performed using R software (http://www.R-project.org, v.4.3.3) with the “TwoSampleMR” package. Figures were generated using the “ggplot2” R package. A *P* value of less than 0.05 was considered statistically significant for associations between exposure and outcome.

**Table 2:** MR result of the association of gut microbiota with HER2+ breast cancer

**Exposure**	**SNPs**	**pval**	**or**	**or_lci95**	**or_uci95**	**Direction**
DAPLYSINESYN-PWY: L-lysine biosynthesis I	9	0.027	1.161	1.017	1.325	Positive
FAO-PWY: fatty acid beta oxidation I	7	0.004	0.842	0.750	0.945	Negative
PWY-4984: urea cycle	8	0.021	0.835	0.717	0.973	Negative
PWY-5005: biotin biosynthesis II	13	0.0179	1.118	1.019	1.226	Positive
PWY0-1298: superpathway of pyrimidine deoxyribonucleosides degradation	11	0.048	0.911	0.831	0.999	Negative
PWY-6147: 6-hydroxymethyl dihydropterin diphosphate biosynthesis I	14	0.016	1.149	1.026	1.286	Positive
PWY-6263: superpathway of menaquinol-8 biosynthesis II	5	0.020	1.116	1.017	1.225	Positive
PWY-6590: superpathway of *Clostridium acetobutylicum* acidogenic fermentation	6	0.015	0.824	0.705	0.963	Negative
Gammaproteobacteria	4	0.038	1.212	1.011	1.453	Positive
Oscillospiraceae	6	0.036	0.837	0.708	0.989	Negative
Pseudoflavonifractor	8	0.006	1.142	1.039	1.256	Positive
Lachnospiraceae noname	5	0.020	1.230	1.033	1.464	Positive
Roseburia	14	0.009	0.857	0.763	0.962	Negative
Haemophilus	5	0.032	0.898	0.813	0.991	Negative
Rothia mucilaginosa	4	0.039	0.904	0.821	0.995	Negative
Parabacteroides merdae	4	0.013	1.244	1.048	1.478	Positive
Pseudoflavonifractor capillosus	10	0.008	1.125	1.032	1.227	Positive
Oscillibacter unclassified	6	0.037	0.837	0.708	0.989	Negative
Bacteroides clarus	9	0.017	1.077	1.013	1.144	Positive

Abbreviations: pval: *P* value; OR: Odds ratio; or_lci95: Odds ratio lower confidence interval at 95%; or_uci95: Odds ratio upper confidence interval at 95%; MR: Mendelian randomization; FAO: Fatty acid β-oxidation.

## Results

### Causal relationship of gut microbiota with breast cancer

Using two-sample MR analysis and the IVW method, this study identified 15 gut microbiota and related pathways significantly associated with breast cancer, including 10 functional pathways and five microbial taxa. As shown in Table 1, six functional pathways and three microbial taxa were positively associated with overall breast cancer. Figure 2 presents the forest plot of bacterial flora positively linked to breast cancer and its subtypes. Breast cancer is a complex disease with diverse molecular and phenotypic backgrounds, resulting in varied clinical outcomes. Although standard breast cancer molecular classifications typically rely on ER/PR/HER2 status, the GWAS data used did not provide corresponding subtype information. Therefore, a subgroup analysis was conducted based on HER2 expression. Causal associations between gut microbiota and HER2+ and HER2- breast cancer were further examined separately using two-sample MR analysis. Table 2 shows that eight functional pathways and 11 microbial taxa were associated with HER2+ breast cancer, including four functional pathways and six microbial taxa positively linked to the HER2+ subtype. Table 3 presents potential causal relationships between 16 gut microbiota and HER2- breast cancer, including five functional pathways and three microbial taxa positively associated with the HER2- subtype.

**Table 3:** MR result of the association of gut microbiota with HER2- breast cancer

**Exposure**	**SNPs**	**pval**	**or**	**or_lci95**	**or_uci95**	**Direction**
METHGLYUT-PWY: superpathway of methylglyoxal degradation	8	0.027	1.125	1.013	1.249	Positive
POLYISOPRENSYN-PWY: polyisoprenoid biosynthesis *E. coli*	8	0.027	0.887	0.797	0.987	Negative
PWY0-1298: superpathway of pyrimidine deoxyribonucleosides degradation	14	0.002	1.252	1.083	1.448	Positive
PWY0-781: aspartate superpathway	11	0.038	1.197	1.010	1.418	Positive
PWY-5005: biotin biosynthesis II	11	0.005	0.848	0.756	0.952	Negative
PWY-6628: superpathway of L-phenylalanine biosynthesis	12	0.021	0.888	0.803	0.982	Negative
PWY-6700: queuosine biosynthesis	15	0.039	0.870	0.762	0.993	Negative
PWY-6892: thiazole biosynthesis I *E. coli*	6	0.018	1.213	1.034	1.422	Positive
PWY-GLYCOLYSIS: glycolysis I from glucose-6-phosphate	15	0.030	0.870	0.767	0.987	Negative
PWY-HEME-BIOSYNTHESIS-II: heme biosynthesis I aerobic	8	0.030	0.829	0.700	0.982	Negative
TRNA-CHARGING-PWY: tRNA charging	12	0.009	1.205	1.048	1.386	Positive
Veillonellaceae	10	0.047	1.141	1.002	1.300	Positive
Butyrivibrio	10	0.021	0.925	0.865	0.988	Negative
Streptococcus thermophilus	4	0.030	0.871	0.768	0.987	Negative
Ruminococcus torques	7	0.015	1.185	1.034	1.358	Positive
Roseburia intestinalis	9	0.034	1.163	1.011	1.337	Positive

Positive: Risk-increasing (OR>1); Negative: Risk-decreasing (OR<1). Abbreviations: pval: *P* value; OR: Odds ratio; or_lci95: Odds ratio lower confidence interval at 95%; or_uci95: Odds ratio upper confidence interval at 95%; MR: Mendelian randomization.

**Table 4:** Heterogeneity, pleiotropy, and sensitivity of total breast cancer

**Exposure**	**Outcome**	**Heterogeneity**		**Pleiotropy**		
		**MR Egger Q (*P* value)**	**IVW Q (*P* value)**	**PRESSO RSSobes (*P* value)**	**Egger_intercept**	**Value of *P***
DAPLYSINESYN-PWY: L-lysine biosynthesis I	Total breast cancer	0.733	0.749	0.771	−0.013	0.435
FAO-PWY: fatty acid beta oxidation I	Total breast cancer	0.433	0.550	0.612	−0.006	0.781
METHGLYUT-PWY: superpathway of methylglyoxal degradation	Total breast cancer	0.368	0.407	0.441	−0.019	0.455
PRPP-PWY: superpathway of histidine purine and pyrimidine biosynthesis	Total breast cancer	0.979	0.938	0.944	0.026	0.199
PWY0-1298: superpathway of pyrimidine deoxyribonucleosides degradation	Total breast cancer	0.923	0.947	0.948	−0.006	0.743
PWY-4984: urea cycle	Total breast cancer	0.138	0.203	0.207	0.011	0.853
PWY-5005: biotin biosynthesis II	Total breast cancer	0.738	0.727	0.737	−0.023	0.352
PWY-6263: superpathway of menaquinol-8 biosynthesis II	Total breast cancer	0.810	0.903	0.904	0.009	0.801
PWY-6590: superpathway of *Clostridium acetobutylicum* acidogenic fermentation	Total breast cancer	0.782	0.709	0.730	0.034	0.336
PWY-7446: sulfoglycolysis	Total breast cancer	0.555	0.642	0.649	−0.008	0.738
Pseudoflavonifractor	Total breast cancer	0.705	0.793	0.800	−0.007	0.774
Lachnospiraceae noname	Total breast cancer	0.336	0.495	0.557	0.003	0.940
Roseburia	Total breast cancer	0.280	0.324	0.362	−0.011	0.568
Parabacteroides merdae	Total breast cancer	0.417	0.364	0.489	−0.066	0.354
Bacteroides intestinalis	Total breast cancer	0.204	0.410	NA	−0.019	0.801

Abbreviations: MR: Mendelian randomization; FAO: Fatty acid β-oxidation; IVW: Inverse Variance Weighted.

### Heterogeneity, pleiotropy, sensitivity, reverse analysis, and BWMR analysis

**Figure 3.**
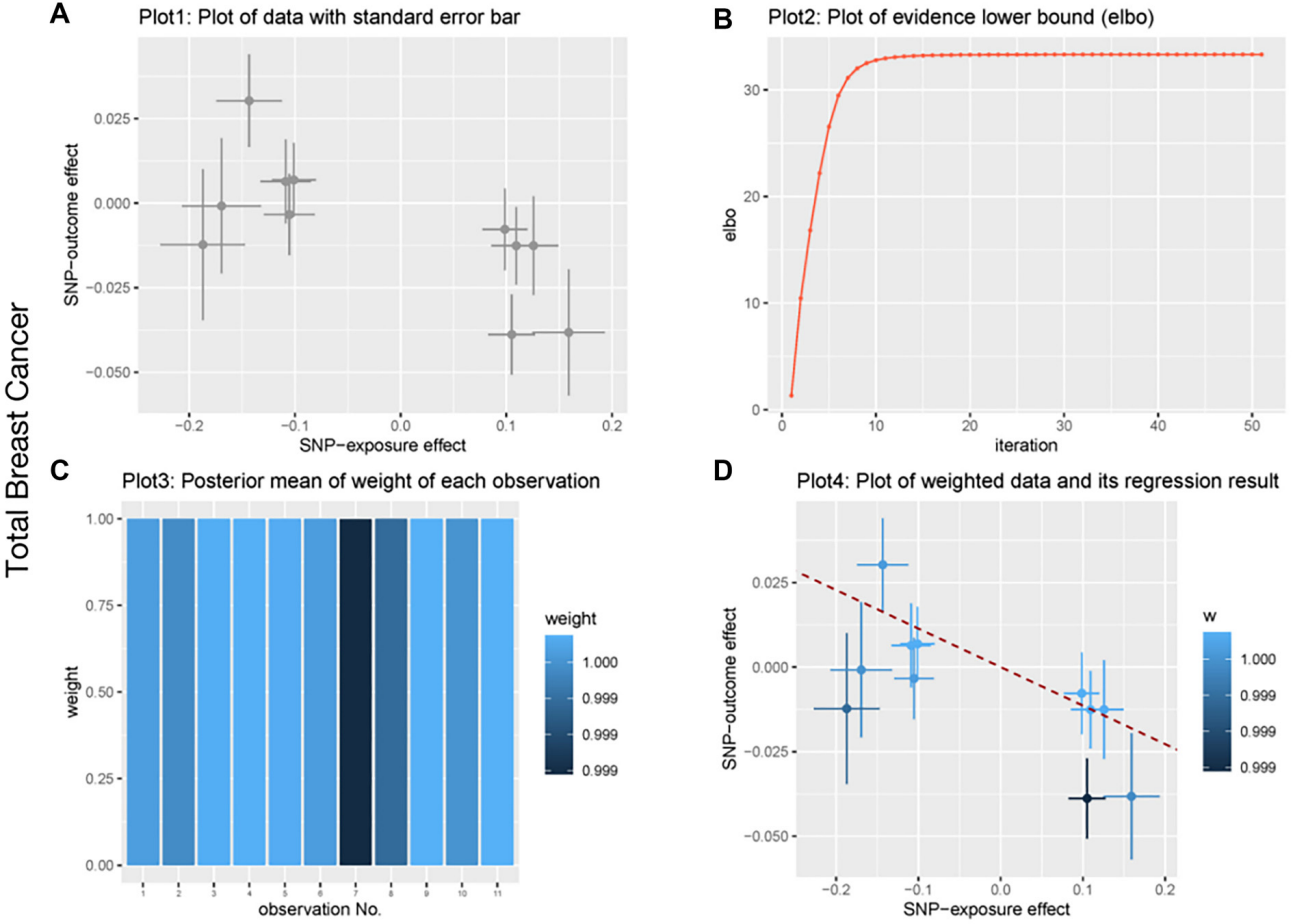
**BWMR analysis of biotin biosynthesis II and breast cancer.** (A) Plot of data with standard error bar; (B) Plot of evidence lower bound; (C) Posterior mean of weight of each observation; (D) Plot of weighted data and its regression result. Abbreviation: SNP: Single-nucleotide polymorphism; BWMR: Biomedical waste management rules.

**Figure 4.**
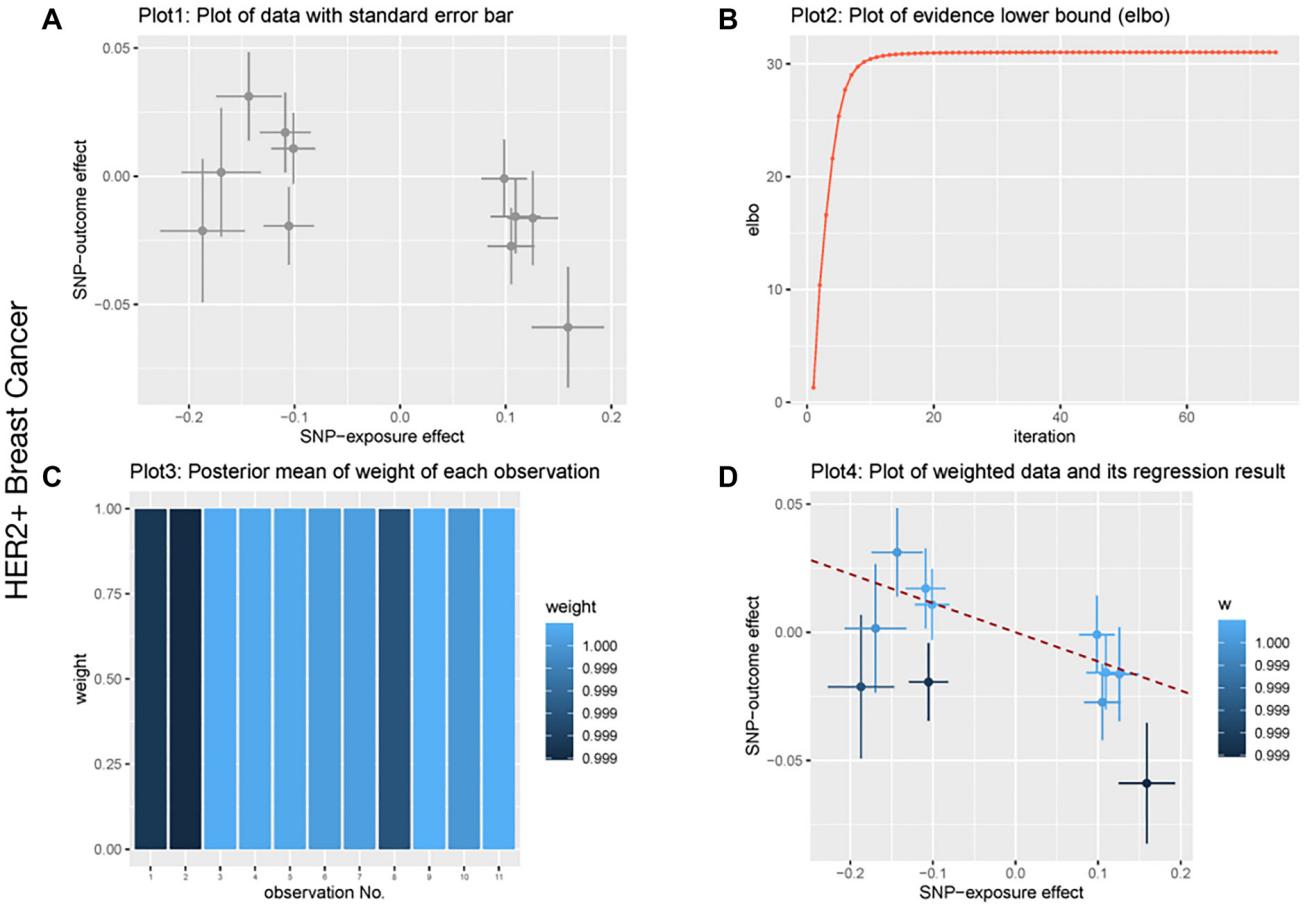
**BWMR analysis of biotin biosynthesis II and HER2+ breast cancer.** (A) Plot of data with standard error bar; (B) Plot of evidence lower bound; (C) Posterior mean of weight of each observation; (D) Plot of weighted data and its regression result. Abbreviation: SNP: Single-nucleotide polymorphism; BWMR: Biomedical waste management rules.

We conducted heterogeneity, sensitivity, and pleiotropy analyses to ensure the robustness of our MR causal effect estimates. The results from the IVW test and MR-Egger regression indicated no heterogeneity in the causal relationship between the gut microbiota and breast cancer, as shown by the Q statistics (*P* > 0.05). Furthermore, the MR-Egger regression intercepts did not significantly differ from zero, suggesting no evidence of horizontal pleiotropy (all intercept *P* values > 0.05). Similarly, the MR-PRESSO test showed no signs of horizontal pleiotropy in the examined causal relationships (*P* > 0.05) (Tables 4–6). No significant heterogeneity or pleiotropy was observed in the analysis of total breast cancer. Additionally, leave-one-out analysis demonstrated that no single SNP disproportionately influenced the causal signals. The funnel plot also supported the reliability of the causal associations identified. In the reverse MR analysis, no supporting evidence was found for a causal effect of breast cancer on gut microbiota. To further validate the association between gut microbiota and breast cancer, we performed a BWMR analysis, which confirmed that the aforementioned gut microbiota were significantly associated (Tables 7–9). The relevant results are visualized in Figures 2–4. Together, these findings suggest a strong causal relationship between specific gut microbiota and breast cancer risk, reinforcing the robustness and reliability of our results.

**Table 5:** Heterogeneity, pleiotropy, and sensitivity of HER2+ breast cancer

**Exposure**	**Outcome**	**Heterogeneity**		**Pleiotropy**		
		**MR Egger Q (*P* value)**	**IVW Q (*P* value)**	**PRESSO RSSobes (*P* value)**	**Egger_intercept**	**Value of *P***
DAPLYSINESYN-PWY: L-lysine biosynthesis I	HER2+ breast cancer	0.352	0.442	0.454	−0.007	0.735
FAO-PWY: fatty acid beta oxidation I	HER2+ breast cancer	0.784	0.864	0.870	0.008	0.778
PWY-4984: urea cycle	HER2+ breast cancer	0.225	0.315	0.359	0.009	0.901
PWY-5005: biotin biosynthesis II	HER2+ breast cancer	0.363	0.453	0.479	−0.004	0.910
PWY0-1298: superpathway of pyrimidine deoxyribonucleosides degradation	HER2+ breast cancer	0.217	0.274	0.316	0.007	0.786
PWY-6147: 6-hydroxymethyl dihydropterin diphosphate biosynthesis I	HER2+ breast cancer	0.804	0.827	0.842	−0.028	0.527
PWY-6263: superpathway of menaquinol-8 biosynthesis II	HER2+ breast cancer	0.605	0.589	0.603	0.038	0.372
PWY-6590: superpathway of *Clostridium acetobutylicum* acidogenic fermentation	HER2+ breast cancer	0.402	0.547	0.582	0.031	0.639
Gammaproteobacteria	HER2+ breast cancer	0.468	0.612	0.616	0.005	0.921
Oscillospiraceae	HER2+ breast cancer	0.679	0.781	0.817	0.002	0.951
Pseudoflavonifractor	HER2+ breast cancer	0.485	0.469	0.534	0.052	0.369
Lachnospiraceae noname	HER2+ breast cancer	0.104	0.129	0.162	−0.013	0.609
Roseburia	HER2+ breast cancer	0.583	0.707	0.683	−0.001	0.979
Haemophilus	HER2+ breast cancer	0.323	0.406	0.498	−0.055	0.553
Rothia mucilaginosa	HER2+ breast cancer	0.670	0.420	0.521	0.066	0.291
Parabacteroides merdae	HER2+ breast cancer	0.875	0.954	0.965	−0.017	0.825
Pseudoflavonifractor capillosus	HER2+ breast cancer	0.834	0.781	0.827	0.028	0.283
Oscillibacter unclassified	HER2+ breast cancer	0.463	0.608	0.615	0.003	0.949
Bacteroides clarus	HER2+ breast cancer	0.424	0.417	0.456	0.030	0.326

Abbreviations: MR: Mendelian randomization; FAO: Fatty acid β-oxidation; IVW: Inverse Variance Weighted.

**Table 6:** Heterogeneity, pleiotropy, and sensitivity of HER2− breast cancer

**Exposure**	**Outcome**	**Heterogeneity**		**Pleiotropy**		
		**MR Egger Q (*P* value)**	**IVW Q (*P* value)**	**PRESSO RSSobes (*P* value)**	**Egger_intercept**	**Value of *P***
METHGLYUT-PWY: superpathway of methylglyoxal degradation	HER2− breast cancer	0.104	0.154	0.188	0.013	0.799
POLYISOPRENSYN-PWY: polyisoprenoid biosynthesis *E coli*	HER2− breast cancer	0.379	0.445	0.467	0.025	0.547
PWY0-1298: superpathway of pyrimidine deoxyribonucleosides degradation	HER2− breast cancer	0.337	0.414	0.436	−0.001	0.984
PWY0-781: aspartate superpathway	HER2− breast cancer	0.132	0.185	0.204	0.002	0.952
PWY-5005: biotin biosynthesis II	HER2− breast cancer	0.778	0.472	0.506	−0.073	0.076
PWY-6628: superpathway of L-phenylalanine biosynthesis	HER2− breast cancer	0.572	0.615	0.626	0.025	0.498
PWY-6700: queuosine biosynthesis	HER2− breast cancer	0.815	0.797	0.810	−0.032	0.318
PWY-6892: thiazole biosynthesis I *E coli*	HER2− breast cancer	0.521	0.525	0.562	−0.042	0.386
PWY-GLYCOLYSIS: glycolysis I from glucose-6-phosphate	HER2− breast cancer	0.967	0.924	0.924	0.043	0.190
PWY-HEME-BIOSYNTHESIS-II: heme biosynthesis I aerobic	HER2− breast cancer	0.401	0.469	0.482	0.026	0.542
TRNA-CHARGING-PWY: tRNA charging	HER2− breast cancer	0.791	0.838	0.884	−0.019	0.657
Veillonellaceae	HER2− breast cancer	0.764	0.837	0.850	0.007	0.881
Butyrivibrio	HER2− breast cancer	0.504	0.563	0.576	−0.018	0.543
Streptococcus thermophilus	HER2− breast cancer	0.613	0.793	0.837	0.016	0.837
Ruminococcus torques	HER2− breast cancer	0.855	0.923	0.939	0.000	0.997
Roseburia intestinalis	HER2− breast cancer	0.369	0.411	0.456	−0.024	0.473

Abbreviations: MR: Mendelian randomization; IVW: Inverse Variance Weighted.

**Table 7:** BWMR analysis of total breast cancer

**Exposure**	**Method**	**or**	**or_lci95**	**or_uci95**	**pval**
DAPLYSINESYN-PWY: L-lysine biosynthesis I	BWMR	1.136	1.032	1.250	0.009
FAO-PWY: fatty acid beta oxidation I	BWMR	0.891	0.805	0.986	0.025
METHGLYUT-PWY: superpathway of methylglyoxal degradation	BWMR	1.061	1.001	1.124	0.046
PRPP-PWY: superpathway of histidine purine and pyrimidine biosynthesis	BWMR	1.123	1.019	1.237	0.019
PWY0-1298: superpathway of pyrimidine deoxyribonucleosides degradation	BWMR	1.154	1.047	1.272	0.004
PWY-4984: urea cycle	BWMR	0.861	0.755	0.982	0.025
PWY-5005: biotin biosynthesis II	BWMR	0.878	0.811	0.951	0.001
PWY-6263: superpathway of menaquinol-8 biosynthesis II	BWMR	1.112	1.026	1.205	0.010
PWY-6590: superpathway of *Clostridium acetobutylicum* acidogenic fermentation	BWMR	0.862	0.753	0.987	0.032
PWY-7446: sulfoglycolysis	BWMR	1.051	1.003	1.101	0.038
Pseudoflavonifractor	BWMR	1.089	1.004	1.182	0.041
Lachnospiraceae noname	BWMR	1.221	1.047	1.423	0.011
Roseburia	BWMR	0.884	0.805	0.971	0.010
Parabacteroides merdae	BWMR	1.191	1.039	1.365	0.012
Bacteroides intestinalis	BWMR	0.908	0.841	0.981	0.014

Positive: Risk-increasing (OR>1); Negative: Risk-decreasing (OR<1). Abbreviations: pval: *P* value; OR: Odds ratio; or_lci95: Odds ratio lower confidence interval at 95%; or_uci95: Odds ratio upper confidence interval at 95%; FAO: Fatty acid β-oxidation; BWMR: Biomedical waste management rules.

### Mediator analysis

We performed a two-sample MR analysis using the IVW method as the primary analytical approach to examine 731 immune cell types and identify those associated with gut microbiota. The selected immune cells were then individually analyzed in relation to total breast cancer, HER2-positive (HER2+) breast cancer, and HER2-negative (HER2-) breast cancer. Our analysis identified several significant immune cells that mediate the effect of gut microbiota on breast cancer. Among functional microbial pathways, PWY-6263: superpathway of menaquinol-8 biosynthesis II was positively associated with total breast cancer. Additionally, DP (CD4+CD8+) % leukocyte was identified as a positive mediator, while Lachnospiraceae noname (a microbial taxon) also showed a positive association with total breast cancer. Conversely, IgD- CD27- % B cell was found to act as a negative (inhibitory) mediator. In HER2+ breast cancer, PWY-6263 again demonstrated a positive association. DP (CD4+CD8+) % leukocyte remained a positive mediator, while IgD- CD27- % B cell continued to act as a negative mediator. Pseudoflavonifractor capillosus showed a positive association with HER2+ breast cancer, whereas CD25ˆhi CD45RA+ CD4 not Treg % T-cell functioned as a negative mediator. In HER2− breast cancer, PWY0-1298: superpathway degradation of pyrimidine deoxyribonucleosides was positively associated, and BAFF-R on CD20- cells acted as a negative mediator. All relevant beta values are reported in Table 10. No other gut microbiota taxa showed associations mediated by immune cells.

**Table 8:** BWMR analysis of HER2+ breast cancer

**Exposure**	**Method**	**or**	**or_lci95**	**or_uci95**	**pval**
DAPLYSINESYN-PWY: L-lysine biosynthesis I	BWMR	1.163	1.025	1.319	0.019
FAO-PWY: fatty acid beta oxidation I	BWMR	0.839	0.741	0.949	0.005
PWY-4984: urea cycle	BWMR	0.852	0.729	0.996	0.044
PWY-5005: biotin biosynthesis II	BWMR	0.907	0.822	1.000	0.049
PWY0-1298: superpathway of pyrimidine deoxyribonucleosides degradation	BWMR	1.149	1.020	1.294	0.022
PWY-6147: 6-hydroxymethyl dihydropterin diphosphate biosynthesis I	BWMR	1.118	1.012	1.235	0.028
PWY-6263: superpathway of menaquinol-8 biosynthesis II	BWMR	0.818	0.691	0.969	0.020
PWY-6590: superpathway of *Clostridium acetobutylicum* acidogenic fermentation	BWMR	1.222	1.006	1.484	0.044
Gammaproteobacteria	BWMR	0.831	0.697	0.992	0.040
Oscillospiraceae	BWMR	1.147	1.036	1.271	0.008
Pseudoflavonifractor	BWMR	1.241	1.029	1.495	0.024
Lachnospiraceae noname	BWMR	0.853	0.753	0.967	0.013
Roseburia	BWMR	0.841	0.710	0.995	0.044
Haemophilus	BWMR	0.894	0.805	0.993	0.037
Rothia mucilaginosa	BWMR	0.901	0.812	0.999	0.049
Parabacteroides merdae	BWMR	1.246	1.055	1.471	0.010
Pseudoflavonifractor capillosus	BWMR	1.131	1.032	1.240	0.009
Oscillibacter unclassified	BWMR	0.832	0.697	0.993	0.041
Bacteroides clarus	BWMR	1.082	1.014	1.153	0.017

Abbreviations: pval: *P* value; OR: Odds ratio; or_lci95: Odds ratio lower confidence interval at 95%; or_uci95: Odds ratio upper confidence interval at 95%; FAO: Fatty acid β-oxidation; BWMR: Biomedical waste management rules.

**Table 9:** BWMR analysis of HER2− breast cancer

**Exposure**	**Method**	**or**	**or_lci95**	**or_uci95**	**pval**
METHGLYUT-PWY: superpathway of methylglyoxal degradation	BWMR	1.126	1.013	1.251	0.027
POLYISOPRENSYN-PWY: polyisoprenoid biosynthesis *E. coli*	BWMR	0.883	0.789	0.989	0.031
PWY0-1298: superpathway of pyrimidine deoxyribonucleosides degradation	BWMR	1.265	1.069	1.497	0.006
PWY0-781: aspartate superpathway	BWMR	1.206	1.010	1.440	0.038
PWY-5005: biotin biosynthesis II	BWMR	0.841	0.743	0.951	0.006
PWY-6628: superpathway of L-phenylalanine biosynthesis	BWMR	0.884	0.795	0.983	0.023
PWY-6700: queuosine biosynthesis	BWMR	0.864	0.752	0.992	0.039
PWY-6892: thiazole biosynthesis I *E. coli*	BWMR	1.221	1.027	1.452	0.024
PWY-GLYCOLYSIS: glycolysis I from glucose 6 phosphate	BWMR	0.869	0.761	0.991	0.036
PWY-HEME-BIOSYNTHESIS-II: heme biosynthesis I aerobic	BWMR	0.823	0.679	0.998	0.047
TRNA-CHARGING-PWY: tRNA charging	BWMR	1.183	1.028	1.362	0.019
Veillonellaceae	BWMR	1.140	1.000	1.298	0.049
Butyrivibrio	BWMR	0.921	0.859	0.989	0.023
Streptococcus thermophilus	BWMR	0.868	0.759	0.993	0.038
Ruminococcus torques	BWMR	1.194	1.043	1.368	0.010
Roseburia intestinalis	BWMR	1.177	1.003	1.381	0.045

Positive: Risk-increasing (OR>1); Negative: Risk-decreasing (OR<1). Abbreviations: pval: *P* value; OR: Odds ratio; or_lci95: Odds ratio lower confidence interval at 95%; or_uci95: Odds ratio upper confidence interval at 95%; BWMR: Biomedical waste management rules.

**Table 10:** Mediation analysis

**Exposure**	**Mediation**	**Total effect (Beta)**	**A (Beta)**	**B (Beta)**	**Indirect effect (Beta)**	**Indirect effect/Total effect**
Total breast cancer (outcome)						
PWY-6263: superpathway of menaquinol-8 biosynthesis II	DP (CD4+CD8+) %leukocyte	0.104	0.259	0.505	0.013	0.126
Lachnospiraceae_noname	IgD− CD27− % B cell	0.192	−0.274	0.052	−0.014	NA
HER2+ breast cancer (outcome)						
PWY-6263: superpathway of menaquinol-8 biosynthesis II	DP (CD4+CD8+) % leukocyte	0.110	0.259	0.076	0.020	0.178
PWY-6263: superpathway of menaquinol-8 biosynthesis II	CD25 on activated Treg	0.110	0.214	−0.061	−0.013	NA
Pseudoflavonifractor capillosus	CD25hi CD45RA+ CD4 not Treg % T cell	0.118	−0.125	0.021	−0.003	NA
HER2− breast cancer (outcome)						
PWY0-1298: superpathway of pyrimidine deoxyribonucleosides degradation	BAFF-R on CD20−	0.225	0.199	−0.076	−0.015	NA

**Figure 5.**
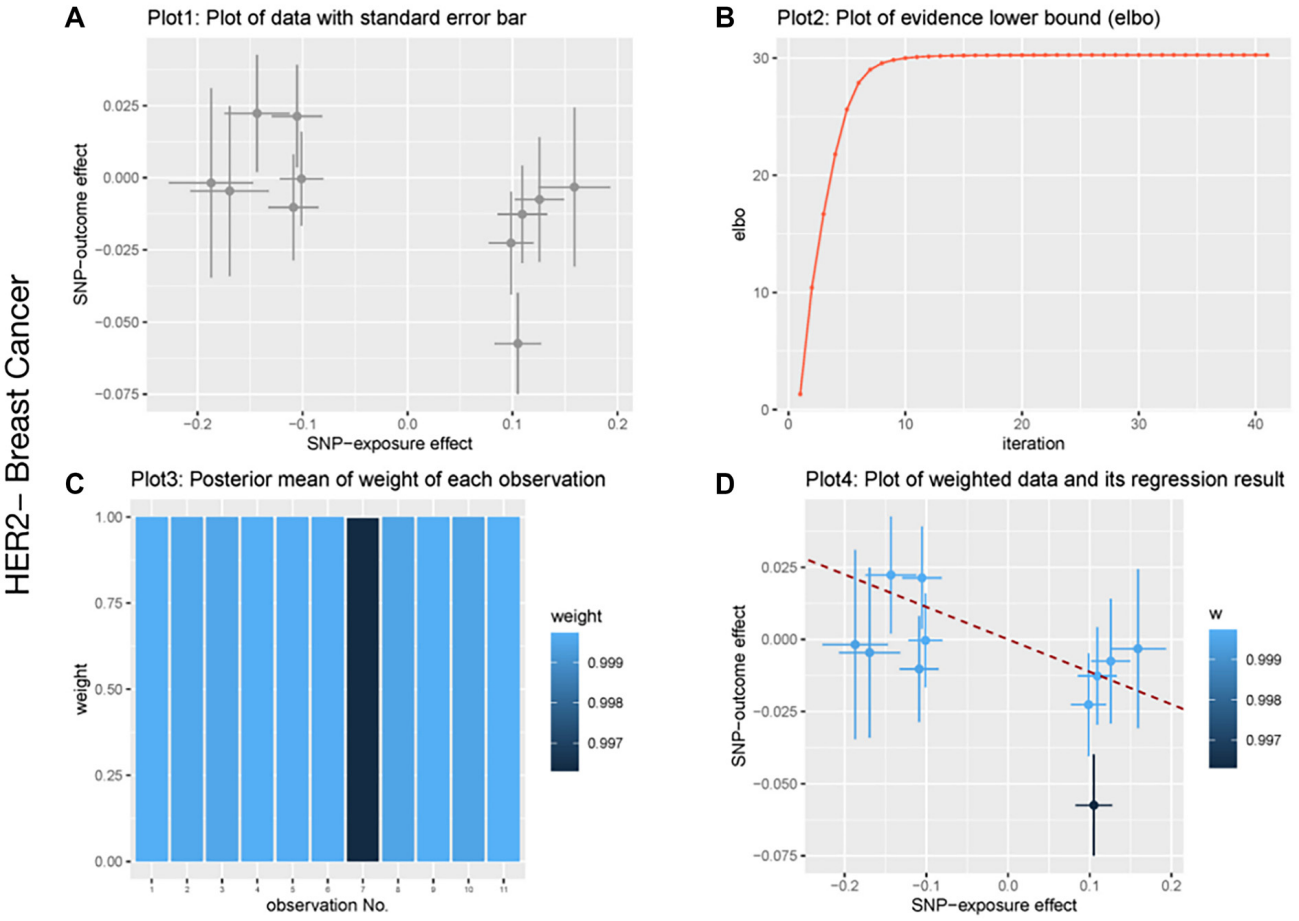
**BWMR analysis of biotin biosynthesis II and HER2− breast cancer.** (A) Plot of data with standard error bar; (B) Plot of evidence lower bound; (C) Posterior mean of weight of each observation; (D) Plot of weighted data and its regression result. Abbreviation: SNP: Single-nucleotide polymorphism; BWMR: Biomedical waste management rules.

**Figure 6.**
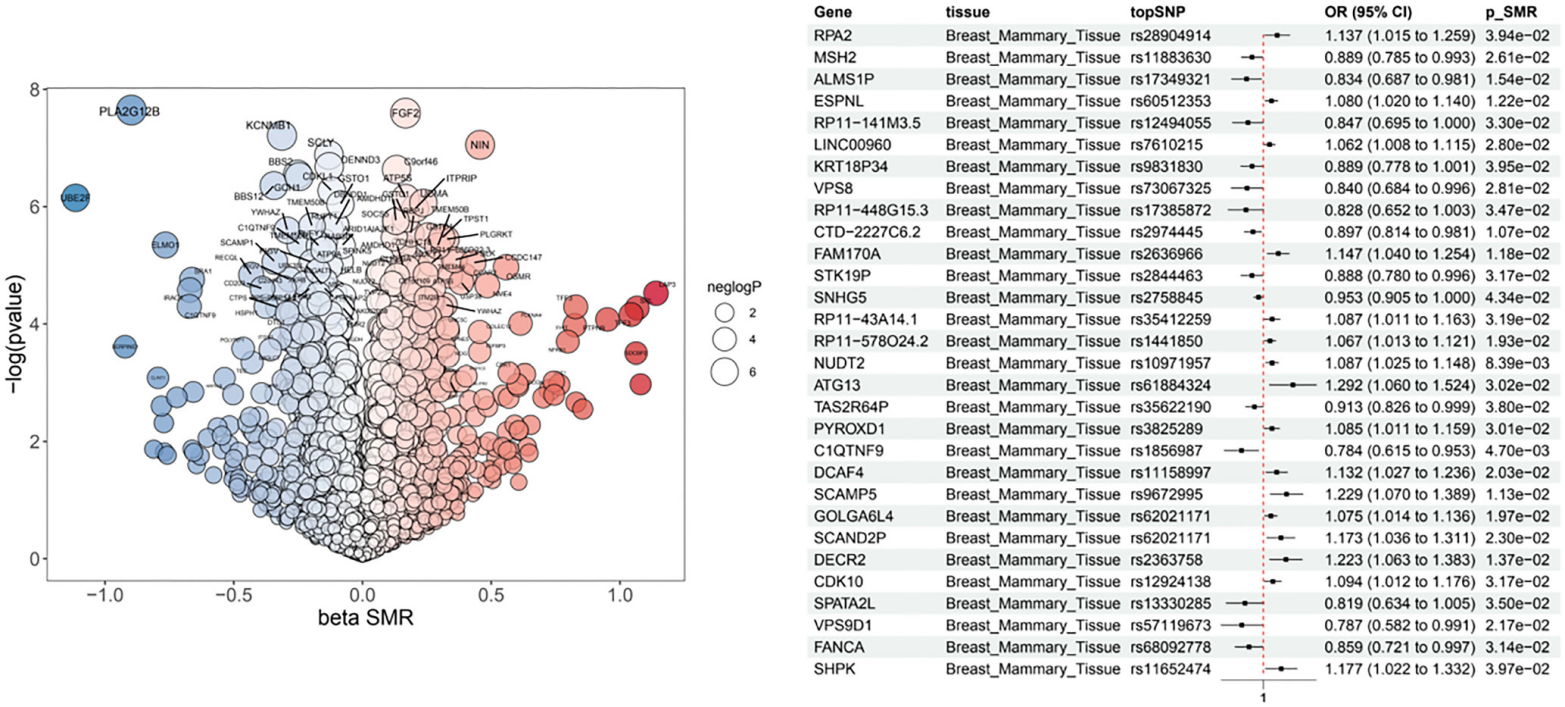
**Genes associated with biotin biosynthesis II in breast cancer**.

### BWMR analysis of biotin biosynthesis II and breast cancer

Considering the important role of biotin biosynthesis II in breast cancer, we further applied BWMR to ensure the robustness of our MR causal effect estimates. The correlations between biotin biosynthesis II and breast cancer are shown in Figures 2–4. Specifically, significant associations were observed for overall breast cancer (*P* ═ 0.03), HER2+ breast cancer (*P* ═ 0.04), and HER2− breast cancer (*P* ═ 0.05). These results further support a causal relationship between biotin biosynthesis II and various breast cancer subtypes.

### Genes associated with biotin biosynthesis II in breast cancer

We further investigated genes related to biotin biosynthesis II in breast cancer. As shown in Figures 5 and 6, 30 associated genes were identified. Among them, RPA2 (OR ═ 1.137, 95% CI ═ 1.015–1.259), ATG13 (OR ═ 1.292, 95% CI ═ 1.060–1.524), and SCAMP5 (OR ═ 1.229, 95% CI ═ 1.070–1.389) were positively correlated with breast cancer. In contrast, MSH2 (OR ═ 0.889, 95% CI ═ 0.786–0.993), ALMS1P (OR ═ 0.834, 95% CI ═ 0.688–0.981), and C1QTNF9 (OR ═ 0.784, 95% CI ═ 0.615–0.953) were negatively correlated with breast cancer.

## Discussion

In our study, we found that 15 gut microbiota were significantly associated with total breast cancer. Among these, six functional pathways and three microbiota taxa were identified as having a promoting effect on the disease. Previous studies have also suggested a potential link between gut microbiota and carcinoma [22–24]. Changes in the immune environment may influence the composition of gut microbiota, thereby contributing to disease development. Numerous recent studies have explored the complex relationship between alterations in gut microbiota, the immune environment, and disease progression. The gut microbiota and immune system share a highly interdependent relationship, and maintaining their balance is essential for overall health. Disruption of this balance can contribute to disease onset [25]. In total, 412 gut microbiota components were evaluated in this study, including 205 functional pathways and 207 microbiota taxa. Of these, 15 gut microbiota were found to have a significant relationship with total breast cancer. Specifically, the pathways FAO-PWY (fatty acid β-oxidation I), PWY-4984 (urea cycle [UC]), PWY-5005 (biotin biosynthesis II), PWY-6590 (superpathway of Clostridium acetobutylicum acidogenic fermentation), as well as the taxa Roseburia and Bacteroides intestinalis, demonstrated protective effects against total breast cancer. In contrast, the remaining associated microbiota were linked to increased risk. Further analysis revealed that among the relevant gut microbiota, seven functional pathways (DAPLYSINESYN-PWY: L-lysine biosynthesis I; FAO-PWY: fatty acid β-oxidation I; PWY-4984: UC; PWY-5005: biotin biosynthesis II; PWY-6263: superpathway of menaquinol-8 biosynthesis II; and PWY-6590: superpathway of Clostridium acetobutylicum acidogenic fermentation) and four microbiota taxa (Pseudoflavonifractor, Lachnospiraceae noname, Roseburia, and Oscillibacter unclassified) were involved in the connection between total breast cancer and HER2+ breast cancer. Additionally, three functional pathways—METHGLYUT-PWY (superpathway of methylglyoxal degradation), PWWY0-1298 (superpathway of pyrimidine deoxyribonucleosides degradation), and PWY-5005 (biotin biosynthesis II)—were implicated in both total breast cancer and HER2− breast cancer. Notably, PWY-5005:biotin biosynthesis II exhibited a protective effect against both total and HER2− breast cancer. Previous research has associated gut microbiota with various diseases, including cancer. Keshet et al. [26] reported that deregulation of the UC metabolic pathway may inhibit cancer progression, as it is the primary mechanism for converting excess nitrogen into excretable urea. Consistent with this, our study found that the functional pathway PWY-4984 (UC) was associated with a reduced risk of breast cancer. Maiti and Paira [27] highlighted the essential role of biotin as a cofactor across all domains of life. Furthermore, certain biotin-targeted Au(I) complexes have shown promise as tumor-targeting radiosensitizers with acceptable safety profiles. Our findings support this by showing a protective role of the gut microbiota pathway PWY-5005: biotin biosynthesis II in total and HER2− breast cancer. Gong et al. [28] found that the DAPLYSINESYN-PWY pathway is associated with obesity, which has been linked to breast cancer [29]. The PWWY0-1298 pathway, which involves the catabolism of pyrimidine nucleotides, such as deoxycytidine, deoxyuridine, and deoxythymidine, may generate important metabolic intermediates through enzymatic degradation [30]. PWY-6263: the superpathway of menaquinol-8 biosynthesis II, likely plays a role in the catabolism of menaquinol-8 (MK-8), a subtype of vitamin K2. MK-8 is essential for spore formation and cytochrome production in certain Gram-positive bacteria [31]. From a chemoprevention perspective, vitamin K2 has been studied for its potential anticancer properties, including the ability to induce autophagy and inhibit cancer cell invasion [32, 33]. Our study supports these findings, demonstrating that the PWY-6263 pathway is protective against both total breast cancer and HER2+ breast cancer.

However, some findings from our study were contrary to previous research. Ma et al. [34] reported that mitochondrial fatty acid β-oxidation (FAO) was a major source of bioenergy that contributed to cancer development. In contrast, our results indicated that fatty acid oxidation I played a protective role. This discrepancy might be due to differences in the gut functional pathways analyzed in our study compared to those in previous FAO-related research, offering new directions for identifying potential therapeutic targets. In our primary MR analysis, we found that PWY-5005 exhibited a negative association with total breast cancer and HER2− breast cancer, but a positive correlation with HER2+ breast cancer. These inconsistent results may stem from the weak association between PWY-5005 and the HER2+ subtype, or from differences in weighting assumptions between TSMR and BWMR methods. The reliability of these findings requires further validation in a larger cohort. Additionally, PWY0-1298, the superpathway of pyrimidine deoxyribonucleosides degradation, appeared to be associated with breast cancer. It showed a positive correlation with total and HER2− breast cancers, but a negative correlation with HER2+ breast cancer. The differing results observed in both IVW and BWMR analyses suggest that this pathway may have only a weak or subtype-specific association, particularly with HER2+ breast cancer. Reverse MR analysis revealed that breast cancer did not significantly impact gut microbiota composition. However, the gut microbiota may influence breast cancer through B cell and T cell-mediated mechanisms involving immune regulation, inflammation, cellular signaling, and metabolic products [35]. Gut microbiota can modulate the maturation and function of T and B cells, potentially promoting tumor growth and metastasis. Moreover, our mediation analysis identified five immune cell types that mediated the relationship between gut microbiota and breast cancer, as well as their specific subsets. These immune cells play essential roles in both innate and adaptive immune responses, orchestrating cellular immunity in cancer and immune disorders. Their coordinated function offers important clinical insights. Gut dysbiosis can lead to overactivation of pro-inflammatory pathways (e.g., NF-κB) and elevated production of cytokines (e.g., IL-6 and TNF-α), thereby fostering a pro-tumorigenic microenvironment. Additionally, gut-derived metabolites, such as short-chain fatty acids (SCFAs) and bile acids can alter immune cell signaling and function through pathways like PI3K-Akt and β-catenin, influencing tumor proliferation and survival [36]. Collectively, these interactions highlight the complex immune-mediated role of gut microbiota in breast cancer development. CD4^+^CD8^+^ double-positive (DP) T cells—a unique subset of T cells—have been found in the blood and peripheral lymphoid tissues of several species. Their involvement in immune diseases, inflammation, and cancer has drawn increasing attention [37]. A previous study found a significant presence of DP T cells in patients with malignant pleural effusion caused by breast cancer metastasis to the thoracic cavity, suggesting a potential role in breast cancer progression [37]. Other research has shown that DP T cells can promote the production of interleukins, such as IL-2 and IL-4, contributing to tumorigenesis and cancer progression [38]. In our study, the proportion of DP (CD4^+^CD8^+^) T cells among leukocytes appeared to mediate the protective effects of PWY-6263, the superpathway of menaquinol-8 biosynthesis II, on total breast cancer and HER2+ breast cancer. These findings further elucidate the potential mechanisms by which gut microbiota may influence tumor initiation and progression. Overall, our MR study explored the relationship between gut microbiota and breast cancer risk. It provided insight into how shifts in intestinal microbiota may lead to immune dysregulation in breast cancer, and offered new strategies for prevention and intervention in its development and progression. However, our study had several limitations. First, our analysis focused exclusively on individuals of European ancestry, excluding other racial and ethnic groups. This limits the applicability of our findings for disease prediction in more diverse populations. In the future, we aim to conduct more detailed analyses that encompass all ethnicities. Second, while molecular subtypes of breast cancer—such as Luminal A, Luminal B, HER2+, and triple-negative—are strongly associated with survival outcomes, the Finnish GWAS dataset used in this study only classified cases into HER2+ and HER2- groups. As a result, we were unable to explore potential causal relationships between gut microbiota and specific breast cancer subtypes. Third, although we implemented methods to identify and remove outliers, including MR-Egger and MR-PRESSO tests, we cannot fully rule out the presence of horizontal pleiotropy or residual population stratification. Horizontal pleiotropy occurs when genetic variants influence the outcome through pathways unrelated to the exposure of interest, violating core assumptions of MR. While approaches like MR-Egger regression, the weighted median method, and the use of negative control outcomes can help mitigate these effects, they have limitations. Similarly, population stratification—caused by differences in allele frequencies and phenotype distributions among subgroups—may introduce confounding that standard adjustments cannot entirely resolve. Although these challenges cannot be completely eliminated, careful study design and advanced analytical techniques can improve the robustness of causal inferences in MR studies. Fourth, we relied on summary-level statistical data, which limited the granularity of our analysis, as individual-level data were unavailable. Further research is needed to investigate additional mediating factors. Finally, because our GWAS data were primarily derived from European cohorts, caution should be exercised when generalizing these findings to other populations, such as those of Asian ancestry. Future studies should aim to replicate these results in more diverse cohorts.

## Conclusion

Our study comprehensively evaluated the relationship among gut microbiota, immune cells, and breast cancer. We found that certain gut microbiota, when considered as exposure factors, may act as either risk or protective factors for breast cancer. Additionally, some immune cells may mediate the effects of gut microbiota on cancer development. These findings offer valuable new insights into the mechanisms by which gut microbiota and immune cells influence breast cancer. However, further experimental and clinical studies are needed to validate and expand upon these results. We also hope our research will contribute to identifying new targets for advancing breast cancer treatment in the future.

## Data Availability

The relevant data are sourced from various published GWAS databases. The data used in this study are publicly available. GWAS data: https://www.finngen.fi/en https://www.ebi.ac.uk/gwas/.
